# Epidemiological study of bovine brucellosis in three senatorial zones of Bauchi State, Nigeria

**DOI:** 10.14202/vetworld.2016.48-52

**Published:** 2016-01-15

**Authors:** S. G. Adamu, N. N. Atsanda, A. O. Tijjani, A. M. Usur, A. G Sule, I. A. Gulani

**Affiliations:** 1 Department of Veterinary Public Health and Preventive Medicine, Faculty of Veterinary Medicine, University of Maiduguri, PMB 1069 Maiduguri, Nigeria; 2 Ministry of Animal and Fisheries Development, Yobe State, Nigeria; 3 Department of Veterinary Medicine, Faculty of Veterinary Medicine, University of Maiduguri, PMB 1069 Maiduguri, Nigeria

**Keywords:** cattle, Bauchi state, brucellosis, Nigeria, seroprevalence

## Abstract

**Aim:**

To determine the seroepidemiological patterns of bovine brucellosis in three senatorial zones of Bauchi State, Nigeria.

**Materials and Methods:**

Blood samples were aseptically collected from the anterior jugular vein of 336 slaughtered cattle, between September 2013 and March 2014 in three senatorial zones of Bauchi State, Nigeria. The sera obtained were screened for brucellosis using rose Bengal plate test (RBPT) and serum agglutination test (SAT) in parallel. The data generated was subjected to Chi-square and Fishers exact test analysis to establish whether there is a relationship between the breeds, sex, and location of the animals sampled.

**Results:**

Of the 336 cattle screened, 18 (5.4%) and 13 (3.9%) were positive by RBPT and SAT, respectively. There was no statistically significant association (p>0.05) between the sex, age, and location of cattle with seropositivity of brucellosis in the state. It was concluded that brucellosis is prevalent in Bauchi State. Further study is recommended in other abattoirs and herds of cattle in Bauchi State for confirmation of the status of the disease among cattle slaughtered in the state.

**Conclusion:**

A high seroprevalence of brucellosis among the cattle in Bauchi state indicates that the disease is endemic and cattle are one of the animals that perpetuate and sustain the disease.

## Introduction

Brucellosis is one of the most common zoonotic diseases in the world. The geographical distribution of brucellosis constantly changes as new foci emerge or re-emerge. The disease occurs worldwide in both animals and humans, except in those countries where bovine brucellosis has been eradicated [1,2]. The disease spread through food and grass contaminated by bacteria, aerosol, broken skin, and mucus membrane contact with the contaminated environment, aborted tissue, fetal fluids, fetal placenta [3,4]. The worldwide economic losses due to brucellosis are extensive, not only in terms of animal production but also in terms of human health. However, when the incidence of brucellosis is controlled in the animal reservoirs, there is a corresponding and significant decline in the incidence in humans [5].

Brucellosis has been reported in African countries [6,7] including Nigeria [8,9]. Factors that may influence the prevalence of brucellosis in Nigeria include management system [10], the herding of different species together [11], use of common ­pastures and water sources [12], age [13], breed [9], sex, lactation status [13], and season [12]. However, other variables such as pregnancy status and state have not been assessed. All these risk factors need to be taken into consideration in designing and execution of effective control programs in Nigeria [14]. Brucellosis infection in animals is endemic in Nigeria, resulting in massive economic losses due to decreased calving percentage, and delayed calving, culling for infertility, the cost of treatment, decreased milk production, abortions stillbirth, the birth of weak calves and loss of man-hours in infected people [15]. The prevalence of brucellosis in cattle might constitute a significant hurdle for the development of livestock in Nigeria. Hence, early and accurate diagnosis is important for undertaking an effective control measure against brucellosis. The serum agglutination test (SAT) and Rose Bengal test are widely used for screening of brucellosis exclusively in eradication programs [16]. The objective was to estimate the seroepidemiology of brucellosis in slaughtered cattle in Bauchi State, to identify strategies for control and eradication of the disease in the country.

## Materials and Methods

### Ethical approval

The experiment was performed according to the care and use of experimental animals’ protocol [17] and was approved by the Faculty of Veterinary Medicine ethics and Research Committee.

### Study area

**Figure-1:**
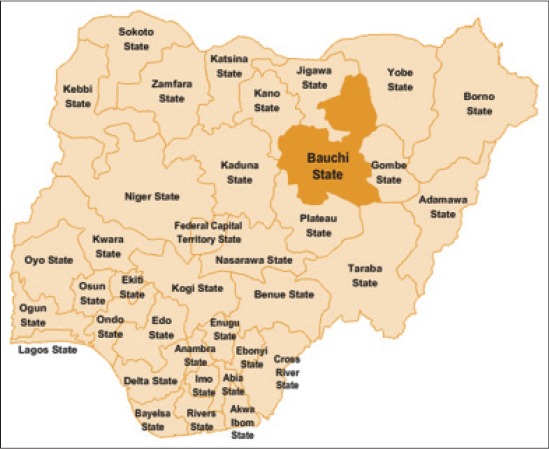
Map of Nigeria showing Bauchi State among the 36 State of the Federation (Source Wikipedia 2014).

**Figure-2:**
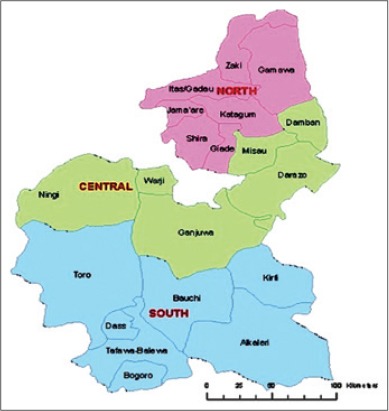
Map of Bauchi State showing the senatorial zones (study area).

Bauchi state (Figure-1) is located between latitude 9°3′ and 12°3′ N, and 8°5′ and 11° E, with a total area of 549,200 km^2^. The state is dry and hot in the north, while the southern part is milder. The rainfall starts in April to October in the southern part, while in the extreme north rains start from late June to September. The state shares boundaries with seven states namely; Kano and Jigawa to the North, Taraba, and Plateau to the South, Gombe and Yobe to the East and Kaduna to the West [18]. The study was carried out in the abattoir of three senatorial zones of Bauchi State (Figure-2); these are Bauchi south, Bauchi central and Bauchi north senatorial zones.

### Sample collection

About 10 ml from each of the 336 cattle slaughtered were aseptically collected from the anterior jugular vein into a sample bottle and each was labeled. The sex, age, breeds, and location of the animals were recorded at the time of blood sample collection. The blood samples were kept in slanted position and allowed to clot, it was then centrifuged at 3000 g for 5 min, and the separated sera were decanted into 2 ml sample bottles (cryovials). The serum samples were then kept at −20°C up to the time of the test.

### Serological test

All sera samples collected were screened in parallel for *Brucella abortus* antibodies using the rose Bengal plate test (RBPT) and SAT techniques as described by Alton *et al*. [19], respectively. The agglutination reactions were recorded as positive (+) or negative (−) depending on whether there were agglutinations or not. Samples with three-fold titer (1:40) and above were confirmed positive for *Brucella* infection. SAT result is less than the RBPT and some are equal. The RBPT antigen was obtained from Indian Veterinary Research Institute, Izatnagar, Uttar Pradesh, India and SAT standard *B. abortus* antigen S99 CVL, New Haw Weybridge, and Surry KT15 3NB, UK. Sera and antigen were taken from the refrigerator and left at room temperature for ½ h before the test, to maintain room temperature and processed following the test procedure recommended by Alton *et al*. [19]. Preparation of the reagent was evaluated by titration and performed according to protocols recommended by World Organisation for Animal Health [20].

The working dilution of antigen for the SAT was a 1:50 dilution of *Brucella* suspension in 0.85% saline. Doubling dilutions of serum were made with saline in tubes, from 1:10 to 1:1280 dilution. High, low, and negative reference sera of known titers were used as controls. Each tube contained 0.5 ml of diluted serum, to which an equal amount of the 1:50 dilution (working dilution) of the antigens was added. The contents of the tubes were mixed, and the tubes incubated in a 37°C shaking incubator for 24 h.

### Statistical analysis

All data generated from this study were analyzed using the Chi-square test with p=0.05 considered as the level of significance.

## Results

A total of 336 cattle screened by RBPT and SAT from the three senatorial zones of Bauchi State, 18 (5.4%) and 13 (3.9%) were seropositive to *Brucella* infection in cattle, respectively. This is comprised 5 (3.0%) and 3 (1.8%) were male while 13 (7.6%) and 10 (5.8%) were female cattle tested by RBPT and SAT respectively (Table-1). There was no statistically significant association (p>0.05) between the sex with seropositivity of cattle brucellosis in the state.

**Table-1:** Seroprevalence of bovine brucellosis in Bauchi State, Nigeria based on sex distribution.

Sex	Number of examined	N (%) positive	

		RBPT	SAT
Male	165	5 (3.0)	3 (1.8)
Female	171	13 (7.6)	10 (5.8)
Total	336	18 (5.3)	13 (3.9)

RBPT=Rose Bengal plate test, SAT=Serum agglutination test

Table-2 shows the age seroprevalence of *Brucella* antibodies in cattle samples that were tested within the state. The highest seroprevalence of 6.3% and 3.9% were obtained in the age band 4-5 years, tested by RBPT and SAT, respectively, followed by 5.5% and 3.7% which was obtained in cattle <4 years. Cattle with the age band 5-7 years, shows 4.6% and 3.7% tested by RBPT and SAT, respectively, while cattle >7 years shows the least seroprevalence of 4.0% for both RBPT and SAT, respectively. There was no statistically significant difference (p>0.05) among all the age bands tested. Based on the location, Bauchi South Senatorial Zone shows the highest seroprevalence of 5.7% and 3.9% by RBPT and SAT respectively, among all the three senatorial zones of the state, followed by Bauchi Central Senatorial zone with 4.7% by both RBPT and SAT respectively, while Bauchi North Senatorial Zone shows the least seroprevalence of 4.5% and 3.0% by RBPT and SAT, respectively. The findings of the study indicated that there was no significant statistical difference (p>0.05) between the rates of infection and location of the cattle in the state (Table-3).

**Table-2:** Seroprevalence of bovine brucellosis in Bauchi State, Nigeria based on age.

Age (years)	Number of examined	N (%) positive	

		RBPT	SAT
<4	54	3 (5.5)	2 (3.7)
4-5	126	8 (6.3)	5 (3.9)
5-7	107	5 (4.6)	4 (3.7)
>7	49	2 (4.0)	2 (4.0)
Total	336	18 (5.3)	13 (3.8)

RBPT=Rose Bengal plate test, SAT=Serum agglutination test

**Table-3:** Seroprevalence of bovine brucellosis in Bauchi State, Nigeria based on location.

Location (senatorial zone)	Number of examined	N (%) positive	

		RBPT	SAT
Bauchi North	66	3 (4.5)	3 (4.5)
Bauchi Central	42	2 (4.7)	2 (4.7)
Bauchi South	228	13 (5.7)	9 (3.9)
Total	336	18 (5.3)	13 (3.8)

RBPT=Rose Bengal plate test, SAT=Serum agglutination test

Based on the breeds of cattle studied, the highest rate of infection was indicated in Bunaji (White Fulani) with seroprevalence of 6.0% and 4.5% by RBPT and SAT, respectively, while Rahaji (red bororo) shows seroprevalence of 4.3% and 2.8% by RBPT and SAT, respectively (Table-4). There was no significant statistical difference (p>0.05) between the two breeds of cattle studied in the state.

**Table-4:** Seroprevalence of bovine brucellosis in Bauchi State, Nigeria based on breeds distribution.

Species	Number of examined	N (%) positive	

		RBPT	SAT
Bunaji (white Fulani)	198	12 (6.0)	9 (4.5)
Rahaji (red bororo)	138	6 (4.3)	4 (2.8)
Total	336	18 (5.3)	13 (3.8)

RBPT=Rose Bengal plate test, SAT=Serum agglutination test

## Discussion

Previous serological surveys in Nigeria showed that the disease is prevalent in different cattle in different rearing areas of the country [9]. A study on cattle husbandry practice in Northeastern part of the country by Adamu *et al*. [9,21] indicated abortion rates and stillbirths, for which brucellosis is more likely to be incriminated. Hence, a cross-sectional study was conducted in three senatorial zone of Bauchi State, to determine the seroprevalence of brucellosis in cattle. In the present study, an overall seroprevalence of 5.3% and 3.8% w recorded in cattle using both RBPT and SAT. This finding is in agreement with the results recorded by Tijjani *et al*. [22] in Yobe with the prevalence of 5.7%, but lower than 36.6% obtained in Adamawa [15] and 34.0% in Yobe [9]. The results obtained in this study are also lower than some results from other African countries, 25.6% in Zimbabwe [7], 8.4% in Sudan [23]. However, the observation of current investigation is higher than 3.5% reported in Ethiopia [24] and 2.77% in Eritrea [2], in which the variation could be due to the difference in sample size used and agroecology. The differences could also be due to variations in animal management and production systems. In the cattle-rearing areas of Nigeria, large numbers of different species of animals are raised on communal pastures and watering areas.

The high and variable seroprevalence of brucellosis has been associated with differences in the specificity and sensitivity of different serological tests in use, large herd size, and extensive movement of cattle and mingling with other herds at common grazing and water point [25].

The seroprevalence of 1.8% obtained in the male was lower than 7.0% in the female cattle tested in this study. The higher seroprevalence among the female than the male cattle studied agreed with earlier reports [13] that the foci of infection remain in females, which spread the infection from one animal to another. The observed higher seroprevalence among the adult age groups than the younger cattle agreed with earlier reports that young animals tend to be more resistant to *Brucella* infection and frequently eliminate the infection while sexually matured animals are more susceptible [13]. The older animals also had higher exposure through sexual transmission. The non-significant statistical difference between the prevalent rate of the senatorial zones and the breeds of cattle studied in the study area may implied that location and breeds of cattle in Bauchi State were probably not the factors affecting the occurrence of brucellosis.

## Conclusion

The result of the present study indicated that brucellosis is endemic among cattle in the studied areas of three senatorial zones of Bauchi State Nigeria, which may represent causes of brucellosis clinical signs such as abortion, enteritis, orchitis, and arthritis. Therefore, screening of cattle both in the herds and abattoir is required before slaughter. Thus, control measures such as vaccination, quarantine, test and slaughter policy and proper disposal of the aborted fetus should be maintained to avoid the spread of brucellosis to human and animals.

## Authors’ Contributions

SGA and NNA: Conception, planning, design, execution and financing of the research, also responsible for collation and analysis of data; preparation of the journal manuscript. AOT: Designed and partly financed and supervised the project, AGS, AMU and IAG: Sample collection, data collation, manuscript vetting and editing. All authors read and approved the final manuscript.
